# Evaluation of Cyclin D1 and Human Epidermal Growth Factor Receptor 2 Neu Protein Expression in Head and Neck Squamous Cell Carcinoma

**DOI:** 10.7759/cureus.35526

**Published:** 2023-02-27

**Authors:** Snigdha Sinha, Subhashish Das, S.M. Azeem Mohiyuddin

**Affiliations:** 1 Pathology, Sri Devaraj Urs Medical College, Kolar, IND; 2 Otorhinolaryngology and Head and Neck Surgery, Sri Devaraj Urs Medical College, Kolar, IND

**Keywords:** lymph node metastases, depth of invasion, head and neck squamous cell carcinoma (hnscc), her2 neu protein, cyclin d1

## Abstract

Background

Head and neck cancers are highly aggressive, frequently occurring cancers that are prevalent worldwide. The mainstay of their treatment is surgery, followed by adjuvant therapy. Various studies have documented the usefulness of molecular markers in carcinogenesis and have proven helpful in the diagnosis and treatment of head and neck cancers. Cyclin D1 is a proto‑oncogene, overexpression of which leads to the accelerated entry of cells in the S phase of the cell cycle, causing uncontrolled proliferation of the cells. The dysregulation of human epidermal growth factor receptor 2 (HER2) neu is also related to multiple features of malignancy, including loss of cell cycle control, induction of angiogenesis, and resistance to apoptotic stimuli. This study seeks to identify a subset of patients with a bad prognosis who may require aggressive treatment strategies.

Aim

This study aims to determine the proportion of the expression of cyclin D1 and HER2 neu in head and neck squamous cell carcinoma (HNSCC) and analyze the association between the expression of cyclin D1 and HER2 neu using histological grading, tumor, node, and metastasis (TNM) staging, and nodal status of the tumor. Furthermore, this study also aims to document clinical outcomes, such as locoregional control, depth of invasion (DOI), and regional metastasis regarding the expression of cyclin D1 and HER2 neu in HNSCC.

Setting and design

This study is a laboratory-based observational study.

Materials and methods

Seventy histologically proven cases of HNSCC were studied for various histopathological parameters, and further immunohistochemistry (IHC) was performed for cyclin D1 and HER2 neu. The expression and intensity of cyclin D1 were multiplied, and the total score was derived. The College of American Pathologists/American Society of Clinical Oncology (CAP/ASCO) guidelines for HER2 neu testing in breast cancer were used for scoring.

Result

Out of 70 cases, 52 (75%) demonstrated strong and moderate positivity for cyclin D1, and the p-values were 0.017, 0.001, and 0.032 for depth of invasion, TNM stage, and lymph node metastases, respectively, for cyclin D1, which was considered statistically significant. For HER2 neu, five out of 70 cases were positive, and the p-value was significant for depth of invasion (0.008).

Conclusion

The expression of the above marker cyclin D1 increases with stage, DOI, and positive lymph node status. Hence, cyclin D1 immunoexpression can be helpful in the early assessment of HNSCC behavior and can serve as an independent prognostic marker. Furthermore, it was observed that HER2 neu was significant with an increase in depth of invasion of tumor, which, in the American Joint Committee on Cancer (AJCC) eighth edition, is considered an important factor for determining the stage of the tumor. Further research is needed to examine whether HER2 neu can act as a prognostic factor for HNSCC and if it can be targeted for treatment options.

## Introduction

Head and neck carcinomas are one of the most common malignancies encountered across the globe. Head and neck carcinoma is a widespread term used for carcinomas involving mainly the oropharynx and upper respiratory tract. The most frequent group among head and neck carcinomas is squamous cell carcinomas (SCCs), comprising nine out of every 10 cases [1]. The prevalence of head and neck squamous cell carcinomas (HNSCCs) is much less in the West than in Asian and South Asian countries with the former accounting for only 1%-4% of the total HNSCC malignancies [2]. Approximately 200,000 cases of HNSCC occur in India every year, which alone accounts for more than 30% of cancer in the country. The majority of the cases in India are found to exist in the oral cavity, especially in the lower gingiva buccal sulcus, which has led to the formulation of the term “Indian oral cancer” [3,4].

Cyclin D1 is strongly associated with the progression of the G1 phase of the cell cycle [5]. When cyclin D1 is overexpressed, the G1 phase is accelerated, and mitogen requirements are reduced [6]. The role of cyclin D1 as an oncogene was established by its ability to cooperate with RAS in cell transformation assays or to complement the defective adenoviral E1a oncogene [7]. The expression of cyclin D1 has been reported in various human tumors of oral, laryngeal, esophageal, and other sites, including breast cancer, mantle cell lymphoma, and squamous cell carcinoma.

The human epidermal growth factor receptor 2 (HER2) protein, also known as ErbB2, is a transmembrane tyrosine kinase receptor. Part of a family of four receptors (ErbB1-4), it influences cancer etiology [8]. The overexpression of HER2 makes it a preferred binding partner for other family members triggering signals that affect cell proliferation, apoptosis, metastasis, and angiogenesis in breast cancer. Due to its unique property, it is a major driver of tumor growth and cancer cell survival [9].

## Materials and methods

This study was conducted in the Department of Pathology with 70 histologically proven resected cases of HNSCC. Hematoxylin and eosin (H&E) slides showing the primary tumor were screened, and the representative blocks were stained to perform immunohistochemistry (IHC) for cyclin D1 and HER2 neu on a 4-mm tissue section. The slides were subsequently deparaffinized and rehydrated through a series of descending concentrations of alcohol for five minutes. A microwave was used for the retrieval of antigen, followed by a distilled water wash and cooling for 10 minutes. Then, 3% hydrogen peroxide was used for blocking peroxidase activity, and incubation was performed with primary antibodies. Subsequently, this was washed thrice with tris buffer solution for five minutes, and a counterstain for hematoxylin was conducted, followed by a tap wash for five minutes. Dehydration and clearing were done with alcohol, xylene, for two minutes. The slides were then mounted with dibutyl phthalate polystyrene xylene (DPX).

Grading of IHC

For the scoring analysis of cyclin D1, each section was observed for five random fields. The nuclear stain was graded on the basis of the expression score, and the intensity scores were calculated by two independent pathologists and further multiplied to give a total score.

The percentage of tumor cells showing nuclear staining for cyclin D1 was graded as follows: 1 = 1%-25% of cells, 2 = 26%-50% of cells, 3 = 51%-75% of cells, and 4 = >76% of cells. The intensity of nuclear staining for cyclin D1 was graded as follows: 1 = mild, 2 = moderate, and 3 = strong. The total score was calculated by multiplying the expression and intensity score, which was graded further as follows: 1-4 = mild, 5-8 = moderate, and 9-12 = strong. For HER2 neu, the College of American Pathologists/American Society of Clinical Oncology (CAP/ASCO) guidelines were followed, and scoring was done as follows: 0 = no membrane staining or staining in <10% of cells, 1 = weak and incomplete staining in >10% of cells, 2 = complete but moderate staining of >10% of cells, and 3 = complete and intense membrane staining of >10%.

Statistical analysis

Data were entered into a Microsoft (MS) Excel data sheet (Microsoft Corporation, Redmond, WA, USA) and analyzed using the Statistical Package for the Social Sciences (SPSS) software version 22 (IBM SPSS Statistics, Armonk, NY, USA). The chi-square test or Fisher’s exact test (for 2 × 2 tables only) was used as the test of significance for qualitative data. Furthermore, survival analysis was performed using the Kaplan-Meier method, and log-rank statistics were used to make comparisons between groups. Univariate survival analysis was carried out using Cox proportional hazards model. A p-value of <0.05 was considered statistically significant after assuming all the rules of statistical tests.

## Results

**Figure 1:**
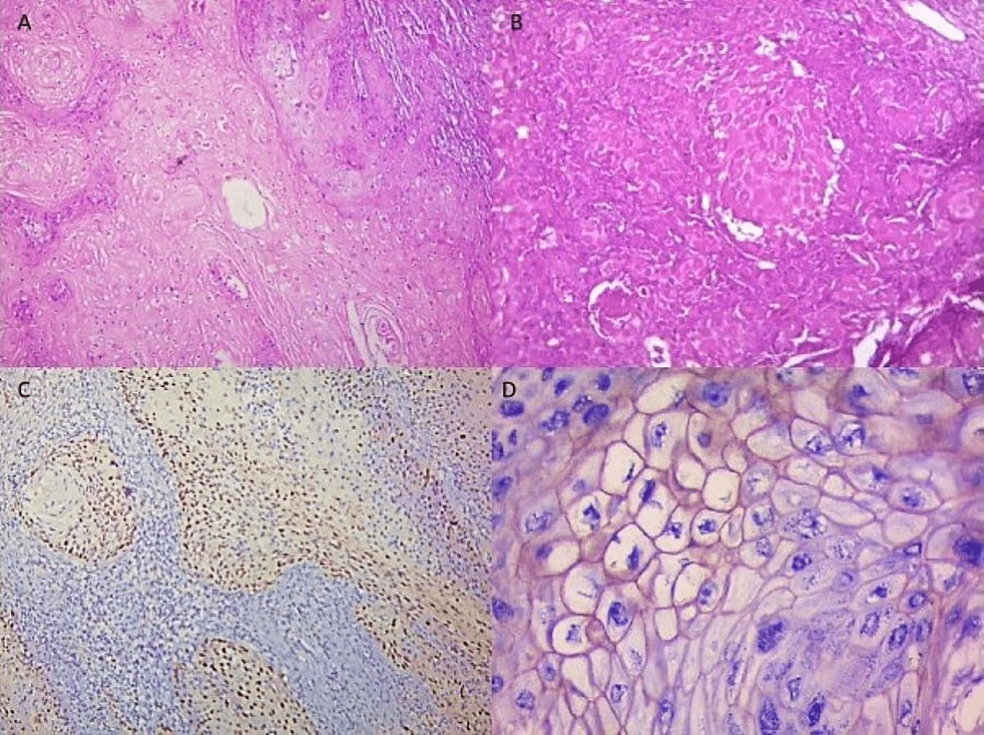
A: Well-differentiated squamous cell carcinoma (hematoxylin and eosin, ×10). B: Moderately differentiated squamous cell carcinoma (hematoxylin and eosin, ×10). C: Strong expression of cyclin D1 immunohistochemistry showing nuclear positivity and expression (3) and intensity (3) (total score: 9) (immunohistochemistry cyclin D1, ×10). D: Positive expression of human epidermal growth factor receptor 2 neu immunohistochemistry showing membranous staining (total score: 2) (human epidermal growth factor receptor 2 neu immunohistochemistry, ×40).

The majority of patients were in their fourth decade of life with females in predominance, and the male-to-female ratio was 1:3. Most of the cases, i.e., 27 out of 70 (38.6%), were in the buccal mucosa, followed by the gingivobuccal sulcus with 17 out of 70 cases. Well-differentiated SCC was observed in the predominant study group with 58 out of 70 cases (82.9%) (Figure 1A). Only 12 (17.1%) cases involved moderately differentiated SCC (Figure 1B). Moreover, the majority of tumors, i.e., 42 out of 70 (60%), had a depth of invasion of less than 20 mm, and a total of 28 out of 70 tumors (40%) had a depth of invasion of more than 20 mm. Based on metastases to the lymph node, the majority of the cases, i.e., 38 out of 70 (54.3%), showed positive involvement of one or more nodes. Furthermore, the staging of carcinomas was done according to the American Joint Committee on Cancer (AJCC) eighth edition. Out of the 70 cases, 43 (61.4%) belonged to stage IV carcinoma and 21 (30%) to stage III. Stage II and stage I had only five cases (7.1%) and one case (1.4%), respectively.

The total score for cyclin D1 was categorized as weak, moderate, and strong. The majority of cases, i.e., 38 out of 70 (54.3%), showed strong positivity for cyclin D1 that demonstrated expression in more than 50% of cells with strong intensity (Figure 1C). Sixteen (22.9%) cases showed moderate and weak scores of cyclin D1 positivity. The p-value for the association of cyclin D1 with the grade of the tumor was 0.157, which was statistically insignificant. Only one case was in the stage I group, which showed weak immunoexpression for cyclin D1. Four out of five (80%) cases in the stage II group showed weak immunoexpression, followed by seven out of 21 (33.3%) cases in the stage III group, which showed weak, moderate, and strong immunoexpression for cyclin D1. For stage IV carcinomas, 31 out of 43 (72.1%) cases were strongly positive for cyclin D1. The p-value was 0.001, and a statistically significant association was found between the stage of the tumor and cyclin D1 immunoexpression. A total of 12 out of 30 (40%) cases of negative lymph node status showed strong immunoexpression for cyclin D1, followed by eight out of 30 (26.7%) and 10 out of 30 (33.3%) cases showing moderate and weak expression, respectively. Furthermore, 26 out of 38 (68.6%) cases showed strong immunoexpression for cyclin D1. The p-value was 0.032, and a statistical significance was found between positive lymph node metastases and cyclin D1 expression (Table 1).

**Table 1:** Association of cyclin D1 expression with various histological parameters. TNM: tumor, node, and metastasis

Parameter		Cyclin D1 expression						p-value
		Weak		Moderate		Strong		
		Total	Percentage	Total	Percentage	Total	Percentage	
Histological grade	Well-differentiated squamous cell carcinoma	13	22.40%	13	22.40%	32	55.17%	0.151
	Moderately differentiated squamous cell carcinoma	3	25%	3	25%	6	50%	
Lymph node metastases	Negative	10	33.30%	8	26.70%	12	40%	0.032
	Positive	4	10.50%	8	21.10%	26	68.40%	
TNM stage	Stage I	1	100%	0	0%	0	0%	0.001
	Stage II	4	80%	1	20%	0	0%	
	Stage III	7	33.30%	7	33.30%	7	33.30%	
	Stage IV	4	9.30%	8	18.60%	31	72.10%	

Only five out of 70 cases were found positive for HER2 expression, showing equivocal positivity (Figure 1D). The majority of the tumors, i.e., 65 out of 70 (92.9%), were negative for HER2 neu expression. All five tumors that were found positive for HER2 neu expression were of more than 20 mm depth of invasion. The p-value was 0.008, and a significant association was found between DOI and HER2 neu expression. No association was established between HER2 expression and TNM staging, histological grade, and lymph node metastases with a p-value of 0.819, 0.932, and 1.00, respectively (Table 2).

**Table 2:** Association of human epidermal growth factor receptor 2 neu expression with various histological parameters. TNM: tumor, node, and metastasis

Parameter		Human epidermal growth factor receptor 2 neu				p-value
		Negative		Positive		
		Total	Percentage	Total	Percentage	
Depth of invasion	<20 mm	42	100%	0	0%	0.008
	>20 mm	23	82.10%	5	17.90%	
Histological grade	Well-differentiated squamous cell carcinoma	23	75.70%	5	7.14%	0.932
	Moderately differentiated squamous cell carcinoma	12	17.14%	0	0%	
Lymph node metastases	Absent	28	93.30%	2	6.70%	1
	Present	35	92.10%	3	7.90%	
TNM stage	Stage I	1	100%	0	0%	0.819
	Stage II	5	100%	0	0%	
	Stage III	20	95.20%	1	4.80%	
	Stage IV	39	90.70%	4	9.30%	

The hazard ratio for overall survival with histopathological parameters was taken out and was significant for the presence of lymph node metastases with a p-value of 0.011, indicating poor overall survival. No association was found with the depth of invasion, primary tumor stage, and histological grade (Table 3). The hazard ratio for disease‑free survival was significant for lymph node status and TNM stage with a p-value of 0.04 and 0.02, respectively (Table 4).

**Table 3:** Hazard ratio for the overall survival of patients with histopathological parameters. CI: confidence interval

Parameter		Hazard ratio	95% CI	p-value
Depth of invasion	<20 mm	0.874	0.327-2.336	0.789
	>20 mm			
Lymph node status	Positive	6.853	1.56-30.100	0.011
	Negative			
Primary tumor	T1-T2	1.446	0.406-5.152	0.569
	T3-T4			
Histological grade	Well-differentiated squamous cell carcinoma	0.431	0.97-1.917	0.268
	Moderately differentiated squamous cell carcinoma			

**Table 4:** Disease-free survival for histopathological parameters. CI: confidence interval, TNM: tumor, node, and metastasis

Parameter		Hazard ratio	95% CI	p-value
Depth of invasion	<20 mm	0.578	0.225-1.48	0.255
	>20 mm			
Lymph node status	Negative	9.03	2.03-4.11	0.004
	Positive			
Primary tumor	T1-T2	0.952	0.320-2.82	0.929
	T3-T4			
Histological grade	Well-differentiated squamous cell carcinoma	0.390	0.088-1.73	0.390
	Moderately differentiated squamous cell carcinoma			
TNM stage	6.17	1.32-28.78	0.020
				>Stage IV

**Figure 2:**
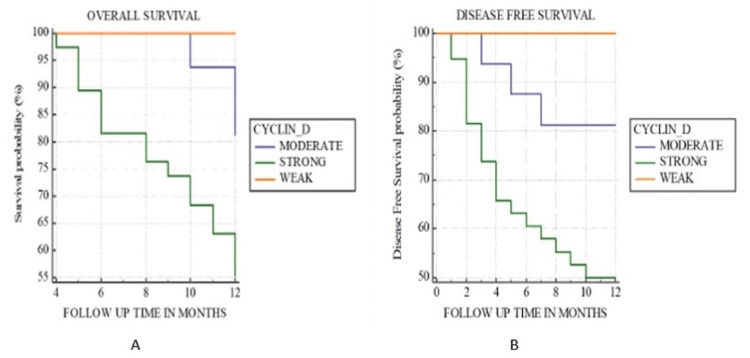
Kaplan-Meier curve for (A) overall survival and (B) disease-free survival of patients with cyclin D1 expression.

**Figure 3:**
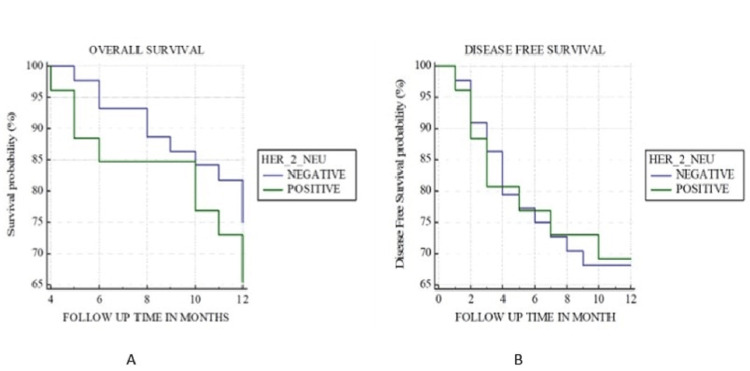
Kaplan-Meier curve for (A) overall survival and (B) disease-free survival with human epidermal growth factor receptor 2 neu immunoexpression. HER2: human epidermal growth factor receptor 2 neu

Furthermore, the Kaplan-Meier curve for the overall survival of patients with cyclin D1 expression indicated poor overall survival with a p-value of 0.02 (Figure 2) and poor disease-free survival with a p-value of 0.008 (Figure 2). No statistical significance was found with the expression of HER2 with overall and disease-free survival (Figure 3).

## Discussion

The mean age for the occurrence of HNSCC in the present study was 53.1 years, which was most consistent with all other studies. The study conducted by Adnan et al. [10] had a mean age of 51.4 years, which correlates with the present study. Similarly, the study conducted by Chinnathambi et al. [11] had a mean age of 56 years, which is also consistent with the present study.

Cyclin D1 immunoexpression was divided as a weak, moderate, and strong expression based on the product after multiplying the intensity and expression score of the marker. In the present study, most of the cases showed strong immunopositivity for cyclin D1, and when combined with moderate expression, the total constituted 54 out of 70 (77.1%) cases. In a study conducted by Siril et al. [12], 46.5% of tumors showed moderate positivity and 26.19% of tumors showed strong positivity, which is in accordance with the present study. Additionally, a study conducted by Menaka et al. [13] showed a maximum number of cases with strong immunoexpression for cyclin D1, i.e., 40% of the entire cases. The study conducted by Dhingra et al. [14] also demonstrated maximum cases with moderate and strong overexpression, i.e., 27 out of 45 cases. All these studies are in accordance with the present study, which further proves that cyclin D1 is expressed strongly in HNSCC. The p-value for the association with the tumor grade was 0.157, which was statistically insignificant. These findings are consistent with the findings of Dhingra et al. [14] and Menaka et al. [13].

A total of 38 out of 70 cases presented with lymph node metastases, out of which 34 cases showed strong and moderate scores for the overexpression of cyclin D1. The result was statistically significant with a p-value of 0.032, proving an association with the overexpression of cyclin D1 in tumors with positive lymph node metastases. Various other studies have also been conducted with the same parameter, and the results obtained were found to be statistically significant. In a study undertaken by Chinnathambi et al. [11], 13 out of 19 (68.4%) cases showed strong and moderate scores for the overexpression of cyclin D1, giving a positive association with the overexpression of cyclin D1 and positive lymph node metastases. Similarly, studies conducted by Huang et al. [15] and Dhingra et al. [14] also showed a positive association with the presence of lymph node metastases, with p-values of 0.002 and 0.008, respectively. All these findings correlate with the findings of the present study, and thus, it can be hypothesized that cyclin D1 expression increases with the presence of positive lymph node metastases.

Moreover, the present study can suppose that cyclin D1 immunoexpression increases with an increase in the stage of the disease. Studies conducted by Zand et al. [16], Lin et al. [17], and Dhingra et al. [14] showed similar results with a p-value of <0.05, thereby demonstrating a positive association between cyclin D1 expression and an increase in the grade of the tumor. On the contrary, the study conducted by Saawarn et al. [18] did not show any association between an increase in stage and the expression of cyclin D1. The p-value for the same was 0.866, and the maximum percentage of cases positive for the expression of cyclin D1 belonged to stage I, with a total case percentage of 66.6%.

In the present study, the majority of tumors (65 out of 70, 92.9%) were found to be negative for the expression of HER2 neu, and only five out of 70 (7.1%) tumors were found to be positive for HER2 neu overexpression. This finding was consistent with all other studies conducted on HER2 neu expression in HNSCC. Studies conducted by Adnan et al. (79 out of 100 tumors, 79%) [10], Vats et al. (56 out of 70 tumors, 80%) [19], and Doley et al. (33 out of 50 tumors, 66%) [20] showed more cases with no immunoexpression of HER2 neu in the patients. The findings of all these studies are consistent with the findings of the present study. Therefore, based on the results, it can be hypothesized that HER2 neu is not expressed in HNSCC.

Additionally, in the present study, the only cases found positive for HER2 neu overexpression were from well-differentiated SCC (five out of 58 cases, 8.62%). The p-value for the association was 0.932, which was statistically insignificant. Other studies conducted by Doley et al. [20] and Rout et al. [21] demonstrated no association between the expression of HER2 neu and the grade of the tumor. On the contrary, the study conducted by Mohanapure et al. [22] showed a positive association with the grade of the tumor, with a p-value of less than 0.05. HER2 neu expression was seen in three out of 38 (7.9%) lymph node-positive cases and two out of 30 (6.7%) lymph node-negative cases. The expression showed no association with the presence of lymph node metastases. Other studies in the literature, including those of Vats et al. [19], Mohanapure et al. [22], and Doley et al. [20], showed similar results with no association between HER2 neu expression and lymph node metastases. All five out of 70 cases that were positive for HER2 neu expression belonged to stage III and stage IV carcinoma, and five out of 64 (7.8%), which is identical to the study conducted by Vats et al. [19], with nine out of 40 (22.5%) cases, were of stage III and stage IV carcinomas. However, no positive association was established between the expression of HER2 neu and the stage of the disease. This finding is consistent with findings in the literature where most of the studies have failed to demonstrate a positive association between the two.

In this study, the follow-up of the patients was conducted for one year, and the association of overall survival was derived with respect to histopathological parameters, cyclin D1 expression, and HER2 neu expression. Furthermore, depth of invasion, lymph node status, primary tumor grade, and histological grade of the tumor were taken as the histological parameters. Univariate survival analysis was done using Cox proportional hazards models. Out of all histopathological parameters taken into consideration, only metastatic lymph node status showed a positive association with overall survival. In the present study, it has been inferred that with a positive metastatic lymph node, the hazard of death increases six times. The p-value for the same was 0.011 and was statistically significant. Other parameters such as depth of invasion, primary tumor grade, and histological grade showed no association with overall survival. The positive association of lymph node status with overall survival correlated with the study conducted by Adnan et al. [10], in which an association was found between metastatic lymph nodes and decreased overall survival, with a p-value of 0.001. Additionally, Cavalot et al. [23] found a positive association between poor overall survival and the presence of metastatic lymph nodes. Based on the above results, an inference can be made that with the presence of metastatic lymph nodes, the overall survival of a patient decreases.

Moreover, in this study, a comparison of overall survival with the expression of cyclin D1 and HER2 neu was made using the Kaplan-Meier curve, and log-rank statistics were used to make a comparison between the two groups. It was observed that patients with strong to moderate expression of cyclin D1 had decreased overall survival. A positive association was present with a decrease in overall survival and strong and moderate expression of cyclin D1, with a p-value of 0.002. A similar result was seen in a study conducted by Lin et al. [17], where the authors stated that an increase in the expression score of cyclin D1 can be associated with poor overall survival. They further hypothesized that cyclin D1 expression influences the survival and prognosis of patients. A similar study by Scantlebury et al. [24] also showed the same result, and statistical significance was found between poor overall survival and cyclin D1 expression.

In the present study, no significant association was derived between the expression of HER2 neu and overall survival. Similar results were seen in studies conducted by Warren et al. [25] and Vats et al. [19]; however, Cavalot et al. [23] suggested that HER2 neu expression can also be helpful in prognosis as an independent risk factor in HNSCC.

Furthermore, histopathological parameters, such as depth of invasion, lymph node status, histological grade, primary tumor grade, and TNM stage, were considered for disease‑free survival. A positive association was observed with the presence of lymph node metastases, with a p-value of 0.004 and a hazard ratio of 9.03, which implied that the presence of metastatic lymph nodes increases the chance of the recurrence of the disease ninefold. Additionally, a positive association was seen with an increase in the TNM stage of the disease, with a p-value of 0.020. It was also observed, from the results, that with an increase in the stage of the disease, the rate of recurrence increases six times. Similar results were seen in the study conducted by Adnan et al. [10], where the p-value was 0.018 and 0.03 for the association of disease-free survival with metastatic lymph node and increase in the TNM stage of the disease, respectively.

It was further observed that patients who showed strong expression of cyclin D1 had poor disease-free survival, and the rate of recurrence was high in such cases. Statistical significance was found for the association of strong cyclin D1 expression score and poor disease-free survival, with a p-value of 0.008. Similar results were noted in a meta-analysis conducted by Ramos-García et al. [26], where statistical significance was found between cyclin D1 expression and poor disease-free survival.

In the present study, no statistical significance was found between disease-free survival and HER2 neu expression. Studies conducted by Adnan et al. [10] and Vats et al. [19] showed no correlation between disease-free survival and HER2 neu expression; this correlates with the results of the present study. On the contrary, Cavalot et al. [23] proved a significant association between HER2 neu expression and poor disease-free survival. They further stated that HER2 neu expression and lymph node-positive status were the only independent variables that were associated with disease-free survival.

Limitations

This is a unicentric study, and the follow-up period was one year. Moreover, the criteria for scoring the expression of human epidermal growth factor receptor 2 neu for head and neck squamous cell carcinoma are not well defined in the literature. Due to this, the result of the present study might differ from the previous other studies.

## Conclusions

Based on the findings of the present study, it can be concluded that cyclin D1 expression increases with an increase in depth of invasion, TNM stage, and lymph node metastases. More importantly, cyclin D1 expression can be associated with poor overall survival and disease-free survival. Hence, based on the above results, cyclin D1 can be used as a prognostic marker in HNSCC.

With respect to HER2 neu, a positive association was seen only with an increase in depth of invasion; no association between HER2 neu expression and overall survival and disease-free survival was observed. Further extensive research is required to understand the role of HER2 neu in HNSCC. Moreover, a standardized system for the scoring of HER2 neu on head and neck cancer is needed. There have been conflicting results on the expression of HER2 neu because of the lack of a standardized scoring system. With further research, HER2 neu may be used for prognostic and therapeutic purposes in HNSCC.

## References

[1] Han AY, Kuan EC, Mallen-St Clair J, Alonso JE, Arshi A, St John MA (2016). Epidemiology of squamous cell carcinoma of the lip in the United States: a population-based cohort analysis. JAMA Otolaryngol Head Neck Surg.

[2] Gupta S, Gupta R, Sinha DN, Mehrotra R (2018). Relationship between type of smokeless tobacco & risk of cancer: a systematic review. Indian J Med Res.

[3] Laprise C, Shahul HP, Madathil SA (2016). Periodontal diseases and risk of oral cancer in Southern India: results from the HeNCe Life study. Int J Cancer.

[4] Sharma S, Satyanarayana L, Asthana S, Shivalingesh KK, Goutham BS, Ramachandra S (2018). Oral cancer statistics in India on the basis of first report of 29 population-based cancer registries. J Oral Maxillofac Pathol.

[5] Orr SJ, Gaymes T, Ladon D (2010). Reducing MCM levels in human primary T cells during the G(0)→4G(1) transition causes genomic instability during the first cell cycle. Oncogene.

[6] Dragoj M, Milosevic Z, Bankovic J, Dinic J, Pesic M, Tanic N, Stankovic T (2015). Association of CCND1 overexpression with KRAS and PTEN alterations in specific subtypes of non-small cell lung carcinoma and its influence on patients' outcome. Tumour Biol.

[7] Natali PG, Nicotra MR, Bigotti A, Venturo I, Slamon DJ, Fendly BM, Ullrich A (1990). Expression of the p185 encoded by HER2 oncogene in normal and transformed human tissues. Int J Cancer.

[8] Maru Y, Hirai H, Yoshida MC, Takaku F (1988). Evolution, expression, and chromosomal location of a novel receptor tyrosine kinase gene, eph. Mol Cell Biol.

[9] Abrahao-Machado LF, Scapulatempo-Neto C (2016). HER2 testing in gastric cancer: an update. World J Gastroenterol.

[10] Adnan Y, Ali SM, Awan MS, Idress R, Awan MO, Farooqui HA, Kayani HA (2022). Hormone receptors AR, ER, PR and growth factor receptor Her-2 expression in oral squamous cell carcinoma: correlation with overall survival, disease-free survival and 10-year survival in a high-risk population. PLoS One.

[11] Chinnathambi PS, Kumar BD (2021). Immunoexpression of cyclin d1 in head and neck squamous cell carcinomas - correlation with histopathological grade and clinical parameters. IP J Diagn Pathol Oncol.

[12] Siril YJ, Kouketsu A, Saito H, Takahashi T, Kumamoto H (2022). Immunohistochemical expression levels of cyclin D1 and CREPT reflect the course and prognosis in oral precancerous lesions and squamous cell carcinoma. Int J Oral Maxillofac Surg.

[13] Menaka TR, Ravikumar SS, Dhivya K, Thilagavathi N, Dinakaran J, Kalaichelvan V (2022). Immunohistochemical expression and evaluation of cyclin D1 and minichromosome maintenance 2 in oral squamous cell carcinoma and verrucous carcinoma. J Oral Maxillofac Patho.

[14] Dhingra V, Verma J, Misra V, Srivastav S, Hasan F (2017). Evaluation of cyclin D1 expression in head and neck squamous cell carcinoma. J Clin Diagn Res.

[15] Huang SF, Cheng SD, Chuang WY (2012). Cyclin D1 overexpression and poor clinical outcomes in Taiwanese oral cavity squamous cell carcinoma. World J Surg Oncol.

[16] Zand V, Binesh F, Meybodian M, Safi Dahaj F, Alamdar Yazdi A (2020). Cyclin D1 expression in patients with laryngeal squamous cell carcinoma. Iran J Pathol.

[17] Lin X, Wen G, Wang S, Lu H, Li C, Wang X (2019). Expression and role of EGFR, cyclin D1 and KRAS in laryngocarcinoma tissues. Exp Ther Med.

[18] Saawarn S, Astekar M, Saawarn N, Dhakar N, Gomateshwar Sagari S (2012). Cyclin d1 expression and its correlation with histopathological differentiation in oral squamous cell carcinoma. ScientificWorldJournal.

[19] Vats S, Ganesh MS, Agarwal A (2018). Human epidermal growth factor receptor 2 neu expression in head and neck squamous cell cancers and its clinicopathological correlation: results from an Indian cancer center. Indian J Pathol Microbiol.

[20] Doley P, Venkataramanappa S, Chikkannaiah P (2020). Significance of human epidermal growth factor receptor 2 neu immunostain in head and neck squamous cell carcinoma. J Sci Soc.

[21] Rout T, Singh A, Epari V (2022). HER 2/neu overexpression in oral squamous cell carcinoma and its clinico-pathological association at a tertiary care center in eastern India. Indian J Otolaryngol Head Neck Surg.

[22] Mohanapure NS, Khandeparkar SG, Saragade PB, Gogate BP, Joshi AR, Mehta SR (2022). Immunohistochemical study of epidermal growth factor receptor, human epidermal growth factor receptor 2/neu, p53, and Ki67 in oral squamous cell carcinoma. J Oral Maxillofac Pathol.

[23] Cavalot A, Martone T, Roggero N, Brondino G, Pagano M, Cortesina G (2007). Prognostic impact of HER-2/neu expression on squamous head and neck carcinomas. Head Neck.

[24] Scantlebury JB, Luo J, Thorstad WL, El-Mofty SK, Lewis JS Jr (2013). Cyclin D1-a prognostic marker in oropharyngeal squamous cell carcinoma that is tightly associated with high-risk human papillomavirus status. Hum Pathol.

[25] Warren EA, Anil J, Castro PD (2021). Human epidermal growth factor receptor 2 expression in head and neck squamous cell carcinoma: variation within and across primary tumor sites, and implications for antigen-specific immunotherapy. Head Neck.

[26] Ramos-García P, González-Moles MÁ, González-Ruiz L, Ruiz-Ávila I, Ayén Á, Gil-Montoya JA (2018). Prognostic and clinicopathological significance of cyclin D1 expression in oral squamous cell carcinoma: a systematic review and meta-analysis. Oral Oncol.

